# Delayed Presentation of a Cervical Spine Fracture: A Case Report

**DOI:** 10.7759/cureus.80705

**Published:** 2025-03-17

**Authors:** Ahmed Mahmood, Moustafa Abouelkheir

**Affiliations:** 1 Accident and Emergency, National Hospital and Medical Center, Lahore, PAK; 2 Emergency, Pilgrim Hospital, Boston, GBR; 3 Community Health Sciences, Fatima Memorial Hospital College of Medicine and Dentistry, Lahore, PAK

**Keywords:** cervical spine fracture, displaced fractures, fracture dislocation of cervical spine, road-traffic accident, spine and trauma, traumatic cervical spine injury

## Abstract

The unique anatomy and flexibility of the cervical spine (C-spine) pose a risk of injury. Trauma to the C-spine encompasses a diverse range of injuries, ranging from minor muscular strains to life-threatening fractures and dislocations associated with spinal cord lesions. This case report concerns a man in his seventies who presented to the emergency department immediately after a road traffic collision with only a minor laceration on his forehead. The patient was discharged after a thorough evaluation and returned the next day, reporting left-hand paresthesia, although the examination yielded no significant findings. This prompted further investigations to confirm multiple cervical vertebrae fractures later.

## Introduction

The cervical spine (C-spine) is a dynamic structure located in the neck that safeguards the nerve innervation extending to the body and facilitates unrestricted movement of the head and neck. However, it is also susceptible to injuries. Cervical spine trauma constitutes a variety of injuries ranging from mild ligamentous or muscular strains to serious fractures or dislocations of bony vertebrae, which can result in significant spinal cord injury. Fractures of the C-spine are a leading cause of mobility loss and fatalities among trauma patients, responsible for 56% of cervical spinal cord injuries and approximately 500 to 600 people enduring acute traumatic spinal cord injury every year in the United Kingdom [1, 2].

Variations in underlying mechanisms such as hyperflexion, hyperextension, axial loading, rotational, and distraction forces predispose the C-spine to injuries [3]. Cervical spine injuries are commonly associated with trauma such as falls, road traffic collisions, sports-related diving injuries, and penetrating or blunt trauma [3]. Nevertheless, non-traumatic C-spine injuries can also occur, such as compression fractures from osteoporosis, arthritis, cancer, or inflammation of the spinal cord [3]. Passias et al. showed that road traffic collisions were the most prevalent cause in the United States, responsible for 29.3% of C-spine fractures and most frequently occurring at the C2 (32.0%) and C7 (20.9%) levels [4].

In some instances, injuries to the C-spine may go unnoticed during the initial assessment or may present symptoms at a later time. Given that the full extent of the injury may not be immediately apparent, it is essential for all patients suspected of C-spine trauma to receive a comprehensive evaluation using a standardized approach. This is vital for enhancing patient outcomes and preventing serious complications, including paralysis and death.

## Case presentation

A 73-year-old male patient presented to the accident and emergency department following a low-speed (20 mph) collision with a stationary vehicle. He was in the front passenger seat wearing his seatbelt and extricated himself after the crash. Upon initial assessment, he had only a 3 cm laceration on the right side of his forehead above the eye, which did not expose bone. He reported no loss of consciousness and was able to recall the entire incident in detail, without any neurological deficit or cervical spine tenderness. Consequently, the National Emergency X-ray Utilization Study (NEXUS) score was determined to be 0. Primary closure of the wound was performed with Prolene 5-0 surgical sutures (Ethicon, part of J&J MedTech, Raritan, NJ) under aseptic conditions and local anesthesia. He was discharged with reassurance and appropriate safety netting, which included monitoring for red flag symptoms indicative of potential neck or head injury, such as paresthesia and paralysis of any limb, severe headache despite adequate analgesia, persistent vomiting, coordination issues, seizures, and lastly, coma.

Following the advice, he returned the next day to the accident and emergency department complaining of left-hand paresthesia. Upon re-assessment, there was no observable tenderness in the cervical spine or any neurological deficits. This included evaluations of deep tendon reflexes, limb strength, limb tone and range of motion, the Glasgow Coma Scale, cranial nerve function, and pupillary size and response. Additionally, he was capable of actively rotating his neck without experiencing any symptoms. The cardiovascular examination, respiratory examination, and abdominal examination were unremarkable. The presence of paresthesia, identified as a red flag symptom and a NEXUS score of one, necessitated further imaging.

The patient’s past medical history included hypothyroidism, type 1 diabetes mellitus, osteoarthritis, anxiety, and depression. He was prescribed levothyroxine 125 mcg, metformin 2 g, pravastatin 40 mg, and citalopram 10 mg to manage these conditions. He had no significant surgical history of note, and his social history consisted of him residing with his partner in their own house.

**Figure 1:**
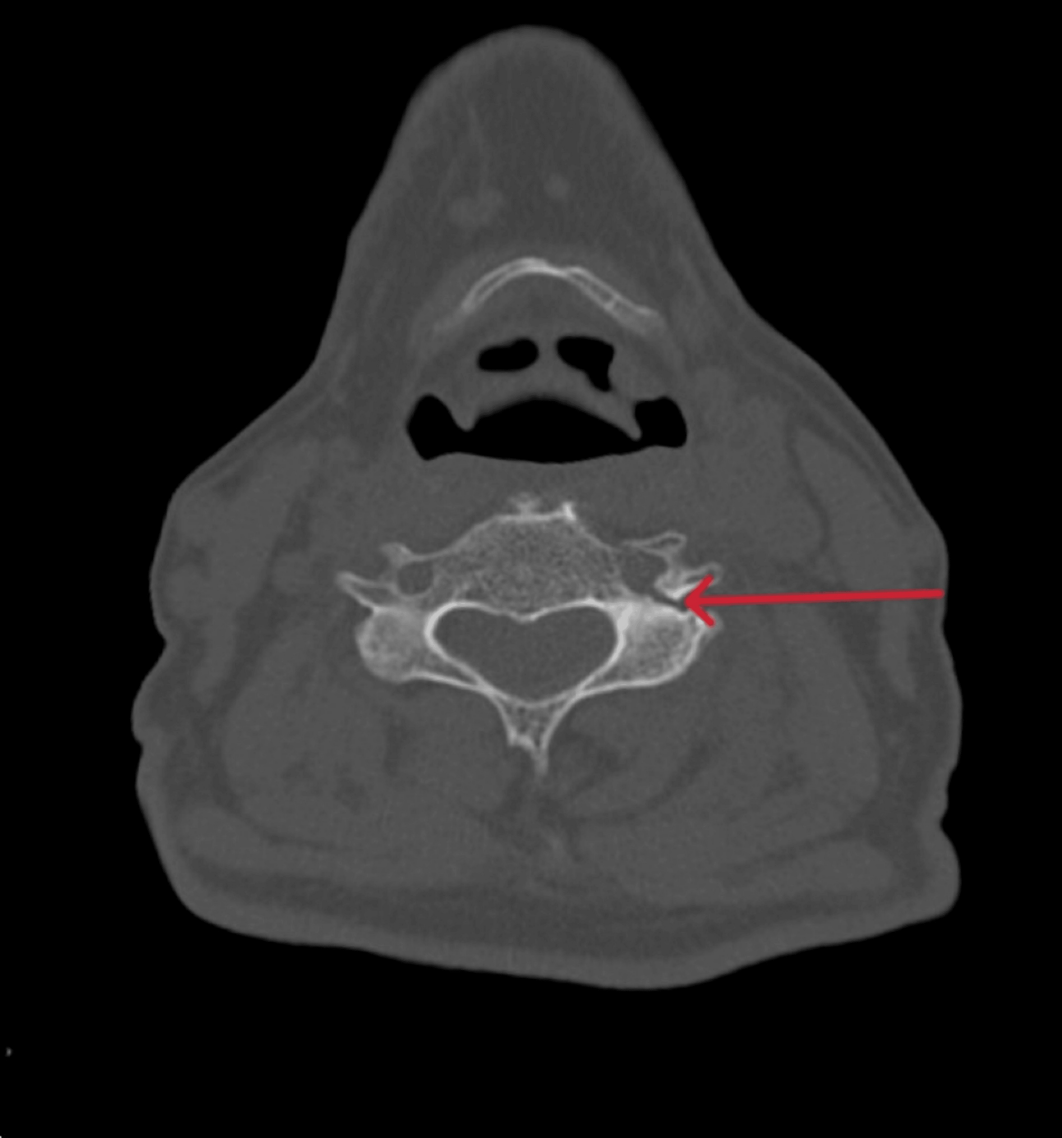
CT scan of the head and cervical spine The red arrow shows a minimally displaced fracture of the left foramen transversarium on an axial view of a non-contrast CT scan of the head and cervical spine.

**Figure 2:**
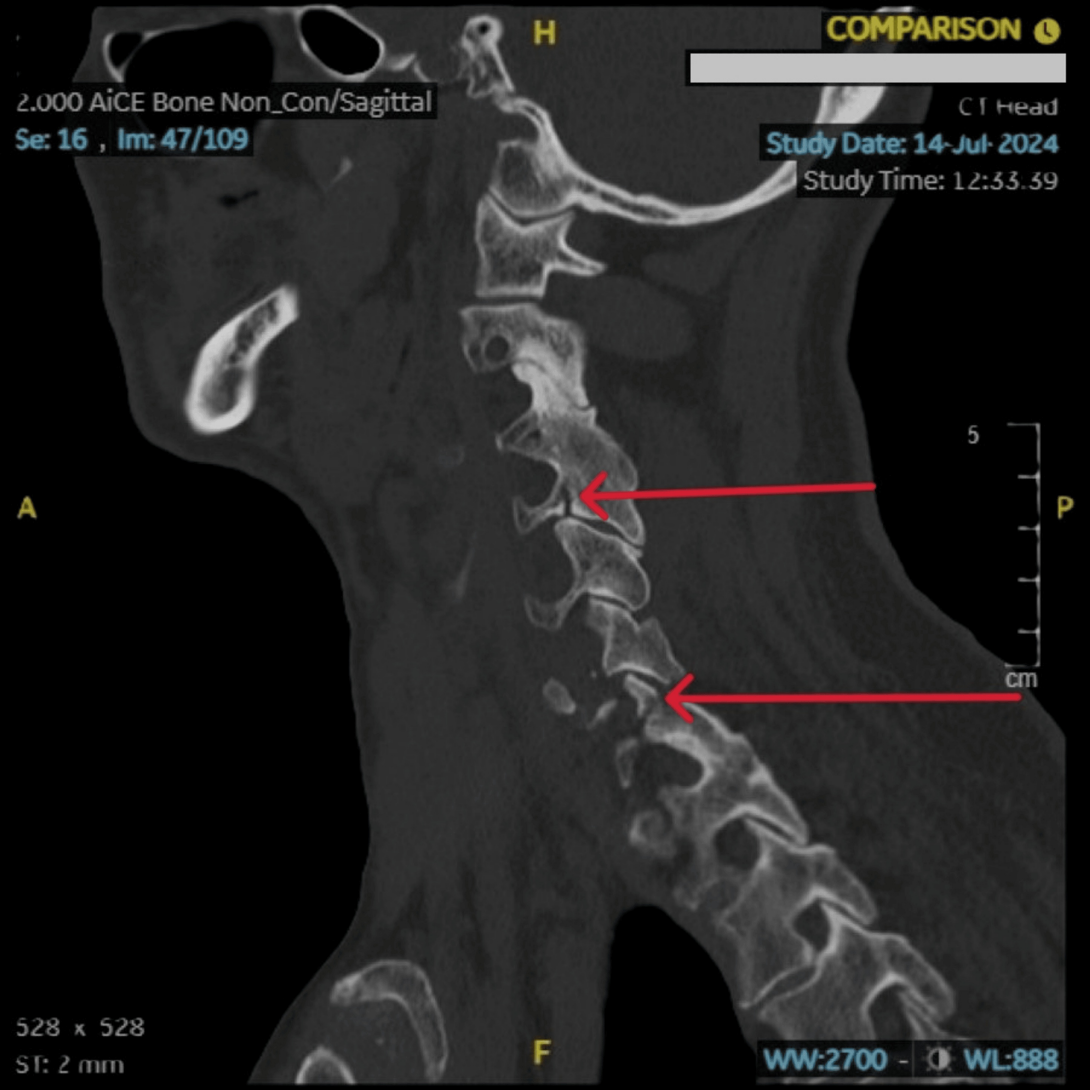
CT scan of head and cervical spine Minimally displaced fracture of the left foramen transversarium of the C4 vertebra and mildly displaced fracture of the left superior articular facet of the C7 vertebra, with minimal diastasis of the left-sided C6-C7 facet joint on a sagittal view of a non-contrast CT scan of the head and cervical spine. These findings are marked by a red arrow on the scan.

**Figure 3:**
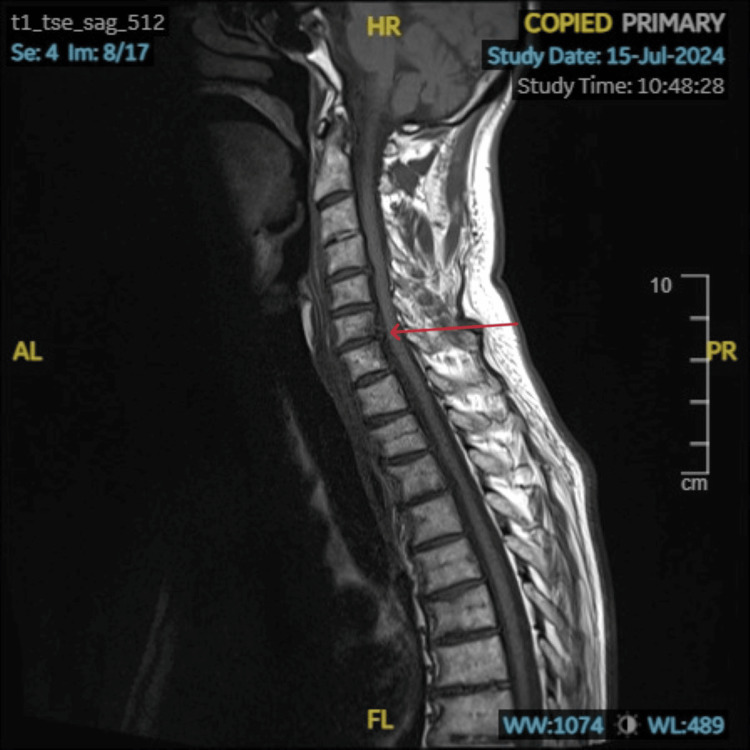
MRI scan of the spine Fracture with surrounding marrow edema/contusion of the left superior articular process at the C7 level and an undisplaced fracture involving the left transverse process at the C7. There is minimal edema involving either aspect of the C6-C7 level intervertebral disc and prevertebral edema in the cervical spine. This is marked with a red arrow.

**Figure 4:**
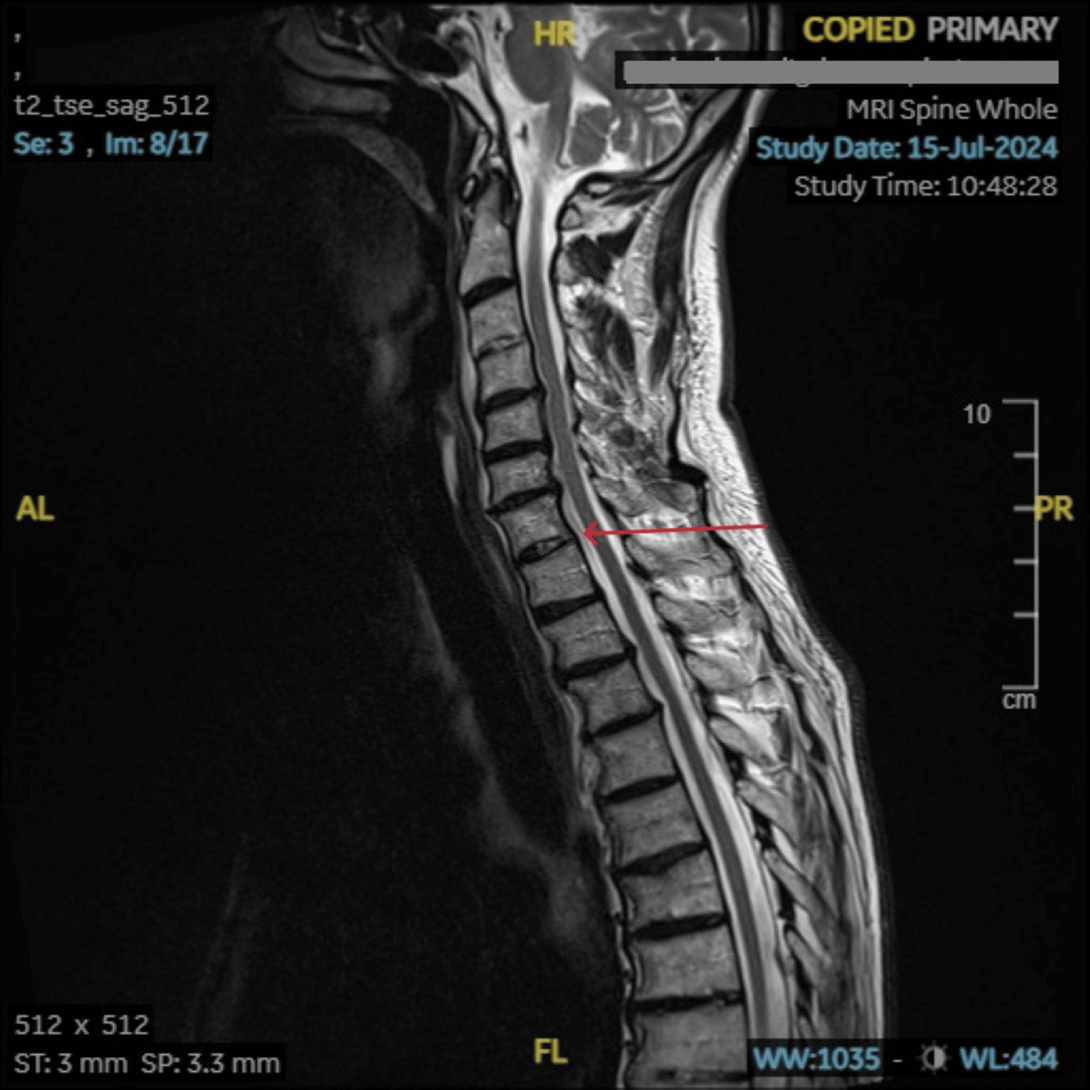
MRI scan of the spine A minimally displaced fracture involving the left transverse process of C4 with involvement of the left transverse foramina is seen. This is marked with a red arrow.

Initial investigations conducted included blood tests (Table 1) and CT scans of the brain and cervical spine (Figures 1, 2, and Table 2). After conducting initial investigations, the patient was referred to the orthopedic team for further evaluation, which included a CT angiogram of intracranial vessels, a CT angiogram of the aortic arch, and an MRI of the whole spine (Figures 3, 4, and Table 2). Following these investigations, a spinal surgery facility at a tertiary care hospital was contacted.

**Table 1:** The patient's blood reports at the time of admission

Parameters	Results	Reference values
Sodium	135	133-146 (mmol/L)
Potassium	4.5	3.5-5.3 (mmol/L)
Urea	3.9	2.5-7.4 (mmol/L)
Creatinine	70	59-104 (umol/L)
Glomerular filtration rate	89	90-200 (mL/min)
Glucose	15.8	3.0-6.0 (mmol/L)
Adjusted calcium	2.48	2.20-2.60 (mmol/L)
C-reactive protein	2.1	0-5 (mg/L)
Hemoglobin	133	132-170 (g/L)
White cell count	6.3	4.3-11.2 10^9 /L
Platelets	4.63	150-400 10^9 /L

**Table 2:** Reports of the patient's radiological investigations including CT scans and MRI of the brain, spine and, arteries

Investigation	Result
CT scan of the brain and spine	No CT evidence of acute intracranial injury/bleeding; Minimally displaced fracture of C4 left foramen transversarium; Mildly displaced fracture of left superior articular facet of C7 vertebra, with minimal diastasis of left-sided C6-7 facet joint; Undisplaced fracture of the left foramen transversarium of the C7 vertebra; Diminished intervertebral disc spaces at multiple cervical levels; No evidence of fracture of the cervical vertebral bodies or post-traumatic collapse
Intracranial CT angiogram	No evidence of arterial occlusion or dissection.
CT angiogram of the aortic arch	No evidence of arterial occlusion or dissection.
MRI of the spine	Degenerative changes involving the spine with disc degenerative changes as described above associated with indentation of the cord at C4-C5 and C5-C6 levels. No definite signal abnormality involving the cervical spinal cord at this level. Fracture with surrounding marrow edema/contusion of the left superior articular process at the C7 level, minimally displaced fracture involving the left transverse process of C4 with involvement of the left transverse foramina, and undisplaced fracture involving the left transverse process at C7 (correlated with CT scan). Minimal edema involving either aspect of the C6-C7 level intervertebral disc and pre-vertebral edema in the cervical spine.

After an initial assessment, the patient was immediately placed on the Aspen® collar (Aspen Medical Products®, Irvine, CA) and referred to the orthopedic team for further management. The orthopedic team contacted the spinal surgery team at a tertiary trauma center, which suggested local admission for observation and conservative management, which included analgesia, hydration, and immobilization of the neck via collar application. The patient was discharged two days following presentation with outpatient follow-up.

## Discussion

The C-spine is composed of seven vertebrae and serves as a protection for the spinal cord. Cervical spine injuries have the potential to cause significant and persistent disability. Cervical spine fractures can be classified based on the level involved and are typically categorized into three groups, namely C1, C2, and the sub-axial spine (C3 to C7) [2]. The sub-axial spine (C5, C6, and C7) represents 55% of C-spine injuries, followed by injury to C2 [3, 5]. The injuries are more prevalent among males and exhibit a bimodal distribution of age, with a prevalence of 15 years to 30 years and above 65 years [3].

Traumatic C-spine injuries can manifest in a diverse range of symptoms, including neck pain, neck stiffness, headache, dizziness, visual symptoms, paresthesia, limb weakness/paralysis, and respiratory compromise in the event of upper cervical fractures [6]. The goal of early detection and management of C-spine injuries, whether surgical or non-surgical, is to return to maximum functional ability, minimize residual pain, reduce any neurological deficit, and prevent further disability. Early detection can be achieved through various scoring systems such as the Canadian C-Spine rule or the NEXUS low-risk criteria (Tables 3, 4) [7, 8].

**Table 3:** The NEXUS C-Spine criteria for the need for radiological imaging NEXUS: National Emergency X-ray Utilization Study Adapted from [7].

Nexus C-Spine criteria	
No posterior midline cervical spinal tenderness	If YES to all, then no radiography is required. If NO to any question then radiography is required.
No evidence of intoxication	
Normal level of alertness	
No focal neurological deficit	
No painful distracting injuries	

**Table 4:** The Canadian C-Spine rule for the need for radiological imaging Adapted from [8].

The Canadian C-Spine rule	
Any high-risk factor? Age at least 65 years or dangerous mechanisms (including falls from at least 1 meter or 5 stairs, axial load to head such as diving, high-speed collision, rollover, ejection from vehicle bicycle collision, motorized recreational vehicles) or paraesthesia in extremities	If yes to any, then needs radiological investigation
Any low-risk factor that allows safe assessment of the range of motion? Simple rear-end motor vehicle collision (excluding rollover, hit by a high-speed vehicle, hit by a large vehicle, pushed into oncoming traffic) or sitting position in an emergency department or ambulatory at any time or delayed onset of neck pain or absence of midline C-spine tenderness	If no to any, then needs radiological investigation
Able to actively rotate neck?	If unable to rotate neck, then needs radiological investigation
If there is no high-risk factor, there is a low-risk factor that allows safe assessment of range of motion and the patient can actively rotate the neck, then there is no need for radiological investigation.	

Different studies have found the Canadian C-spine rule is superior to the NEXUS low-risk criteria for better assessment of cervical spine injury [9]. Hence, the National Institute for Health and Care Excellence (NICE) and the Royal College of Emergency Medicine (RCEM) recommend utilizing the Canadian C-spine rule over NEXUS low-risk criteria [1, 10]. When a C-spine fracture is suspected, it is important that the patient be immobilized and urgently investigated using different radiological modalities as per NICE guidelines [1].

The treatment of cervical fractures varies based on the level and extent of injury. However, it can broadly be categorized into two distinct systems, namely upper cervical spinal injuries comprising C1, C2, and sub-axial spinal injuries comprising C3-C7 [11, 12]. Since the patient had a sub-axial cervical spinal fracture, largely only its management will be discussed. The Sub-axial Injury Classification and Severity Scale (SLICS) is used to assess and determine whether surgical correction or conservative management is necessary (Table 5) [2, 13].

**Table 5:** Sub-axial Injury Classification and Severity Scale (SLICS) for further management of sub-axial cervical spine fractures Adapted from [2, 13]

Sub-axial Injury Classification and Severity Scale (SLICS)			
Category	Parameter	Description	Points
1	Injury morphology	Compression	1
		Burst	2
		Distraction	3
		Rotation/translation	4
2	Disco-ligamentous complex integrity	Intact	0
		Suspected disruption	1
		Disruption	2
3	Neurological status	Intact	0
		Nerve root injury	1
		Complete cord injury	2
		Incomplete cord injury	3
		Persistent cord injury	+1
Management plan according to points			
1-3 points		Non-surgical management	
4 points		Surgical or non-surgical management	
5-10 points		Surgical management	

The occurrence of missed or delayed C-spine injuries ranges from approximately 4.9% to 20% following the initial trauma assessment [14, 15]. The primary factors contributing to these missed or delayed diagnoses include insufficient or incomplete neurological evaluations, inadequate imaging or errors in interpreting the imaging results, and the existence of distracting injuries [10, 14, 15]. To avoid the risk of missing cervical spine injuries, various standardized scoring systems have been implemented, including the Canadian C-Spine Rule and NEXUS low-risk criteria [7, 8].

On the initial presentation, the NEXUS score was 0. However, subsequent presentation to the accident and emergency department revealed a NEXUS score of one, which necessitated radiological investigations, specifically a CT scan, due to its high priority as the gold standard investigation [16]. The CT scan revealed multiple sub-axial C-spine fractures, prompting the spinal team at the tertiary hospital to request an additional MRI scan to determine the severity as per the SLICS scoring system. The patient had a SLICS score of two, which reflected non-surgical or conservative management.

## Conclusions

The objective of this case is to recognize the importance of early recognition of cervical spinal injuries, keeping a low threshold for investigating patients as these injuries are potentially serious and can lead to devastating consequences if not properly treated. Moreover, implementing a standardized protocol is crucial to prevent missing a C-spine fracture. Additionally, it is imperative that a multidisciplinary team approach be utilized as early as possible. This involves collaboration between emergency physicians, radiologists, orthopedic surgeons, spinal surgeons, nursing staff, and paramedics to provide comprehensive management to reduce patient mortality, morbidity, and disability.
